# Correspondence: Reply to: ‘Correspondence: Variations in ocean heat uptake during the surface warming hiatus'

**DOI:** 10.1038/ncomms12542

**Published:** 2016-08-19

**Authors:** Wei Liu, Shang-Ping Xie, Jian Lu

**Affiliations:** 1 Scripps Institution of Oceanography, University of California San Diego, La Jolla, California 92093, USA; 2 Pacific Northwest National Laboratory, Richland, Washington 99354, USA

Nature Communications 7:12542 doi: 10.1038/ncomms12542 (2016); Published 08 19 2016

Energetics arguments are often invoked to explain the early 2000s slowdown of the global mean surface temperature (GMST) increase, a phenomenon often dubbed as the ‘hiatus'. Our recent paper^1^ showed that in climate models, there is no significant difference in global ocean heat uptake during the hiatus and surge (accelerated warming) events^2^, but we identified a vertical redistribution of heat in the ocean^3^. In comments on our paper, Chen and Tung^4^ insist that the horizontal redistribution of subsurface ocean heat content (OHC) is somehow tied to global mean surface temperature variations. But the mechanism for such a relationship has never been demonstrated with the exception of the wind-induced upper ocean adjustments in the tropical Indo-Pacific Oceans^5^,^6^,^7^.

Chen and Tung^4^ suggest that models underestimate inter-decadal variability in ocean heat uptake. Indeed, ocean heat uptake estimates based on ocean temperature observations are more variable in time than in climate models, but the model results are consistent with satellite-based radiative imbalance at the top of the atmosphere (TOA)^8^,^9^. Specifically, since 2000 during which reliable global radiative observations are available, TOA radiative imbalance remains largely unchanged, whereas the rate of estimated OHC change shows much larger temporal variations^9^ (Fig. 1). Since the TOA radiative imbalance approximately equals global ocean heat uptake on inter-annual and longer timescales, this illustrates the limitations of using historical ocean observations to quantify interannual–decadal variations of ocean heat uptake. Given limitations of observational estimates as well as model simulations, the synthesis of observations and models for physical consistence is the best way forward. Blindly trusting models is dangerous, but the critical evaluation of observational estimates is equally important, especially with sparsely sampled measurements of subsurface ocean temperature.

In other technical comments, Chen and Tung^4^ claim that our interpretation of the observational results on the relationship between inter-basin redistribution of 300–1,500 m OHC and the surface warming rate is inappropriate. Specifically, they object to the comparison of 300–1,500 m OHC trends during 1998–2012 against the baseline of 1970–2012, and question our analysis that measures heat redistribution with the basin to global heat uptake ratio. We disagree. In light of large uncertainties in OHC observations (Fig. 2), the large increase of global OHC trend during 1998–2012 in the Ishii data is questionable. In the climate model we analysed, changes in 300–1,500 m OHCs of the global, Atlantic and Southern Oceans are dominated by anthropogenic warming and are not unique to the surface warming hiatus period of 1998–2012. This justifies our use of 1970–2012 as the baseline to isolate anthropogenic warming and supports our analysis of heat redistribution to reveal the dominance of the Atlantic and Southern Oceans in subsurface ocean storage of anthropogenic heat.

## Methods

### Incoming radiation data

The net incoming radiation at the TOA is obtained from the Diagnosing Earth's Energy Pathways in the Climate system (DEEP-C) reconstructed TOA radiative energy fluxes^10^,^11^. This reconstructed data is based on Clouds and the Earth's Radiant Energy System (CERES) Edition 2.8 product but extended from January 1985 to May 2015. A geodetic weighting is used for accurately estimating the global and hemispheric energy imbalance. The reconstructed data is globally covered with monthly outputs. Information of other data can be found in our original paper^1^.

### Data availability

The DEEP-C reconstructed TOA radiative energy fluxes can be found at http://www.met.reading.ac.uk/~sgs02rpa/research/DEEP-C/GRL/.

## Additional information

**How to cite this article:** Liu, W. *et al*. Reply to: ‘Correspondence: Variations in ocean heat uptake during the surface warming hiatus'. *Nat. Commun.* 7:12542 doi: 10.1038/ncomms12542 (2016).

## Figures and Tables

**Figure 1:**
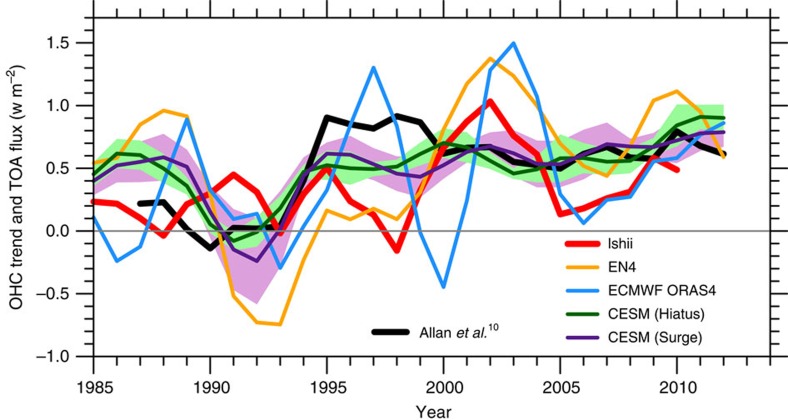
Energy budget. The five-year running mean global ocean heat content (OHC) trend and net incoming radiation at the TOA from satellites^10^,^11^ (black). The OHC trend is expressed as a flux relative to the total surface area of the Earth and calculated from three observational datasets: the Ishii data^12^ (red, 0–1,500 m), the EN4 data^13^ (orange, full depth) and the European Centre for Medium-Range Weather Forecasts (ECMWF) Ocean Reanalysis System 4 (ORAS4) data^14^ (blue, full depth) and the Community Earth System Model (CESM) simulations^15^: the Hiatus/Surge group^1^ (dark green/purple curve for the ensemble mean and light green/plum shading for ±1 s.d. range across ensemble members). Annual mean time series are used in the calculation. The methodology for computing the OHC trend and TOA flux follows Smith *et al*.^9^ The year represents the centre of the time period used to calculate the trend.

**Figure 2:**
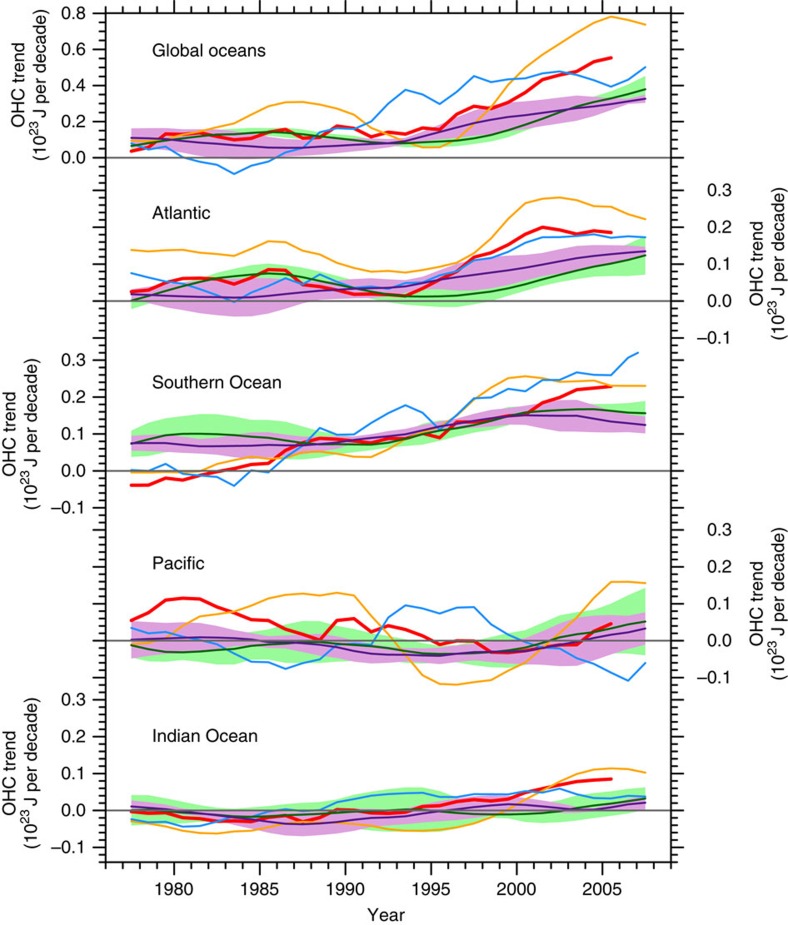
300–1,500m ocean heat content trends. The 15-year running mean 300–1,500 m ocean heat content (OHC) trends in the global oceans, Atlantic, Southern Ocean, Pacific and Indian Ocean from observations: the Ishii (red), EN4 (orange) and ECMWF ORAS4 data (blue), and the CESM simulations: the Hiatus/Surge group (dark green/purple curve for the ensemble mean and light green/plum shading for ±1 s.d. range across ensemble members). Annual mean time series are used in the computation. The year represents the centre of the time period used to calculate the trend.
